# Stem cell-based test methods

**DOI:** 10.17179/excli2019-1550

**Published:** 2019-06-24

**Authors:** Florian Seidel

**Affiliations:** 1 Leibniz Research Centre for Working Environment and Human Factors

## ⁯⁯

***Dear Editor,***

Recently, Agapios Sachinidis and colleagues from the University of Cologne published a review about possibilities and limitations of stem cell-based test methods in pharmacology and toxicology (Sachinidis et al., 2019[18]). In recent years, much progress has been achieved concerning *in vitro* techniques of liver (Godoy et al., 2013[7]; Grinberg et al., 2014[10]; Leist et al., 2017[15]; Ghallab et al., 2016[6]), kidney (Sjögren et al., 2018[24]; Jiang et al., 2018[11]; Su et al., 2016[25]; Valente et al., 2012[26]; Lee et al., 2017[14]), neuronal (Keil et al., 2018[12]; Yang et al., 2018[28]; Colaianna et al., 2017[5]; Sisnaiske et al., 2014[23]) and developmental toxicity (Adam et al., 2019[2]; Bridges et al., 2019[3]; Abbott, 2019[1]). Particularly, in developmental and reproductive toxicity testing, large numbers of animals are needed for analysis of a single compound (Krug et al., 2013[13]). Therefore, stem cell-based test systems are currently developed (Shinde et al., 2014[19], 2015[21], 2016[22]; Krug et al., 2013[13]; Meganathan et al., 2012[16]). They recapitulate differentiation into cells of the three germ layers (Meganathan et al., 2012[16]; Shinde et al., 2016[22], 2017[20]) or differentiation into neural ectodermal progenitor cells (Rempel et al., 2015[17]; Waldmann et al., 2014[27]). Stem cells are exposed to test compounds during the differentiation process and compound associated gene expression changes are monitored. 

Indices have been developed to identify a possible hazard of developmental toxicity based on genome-wide expression data. A precondition is the definition of so-called developmental genes of a test system. Developmental genes are up- or down-regulated during the differentiation process in the absence of test compounds. Developmental potency describes the fraction of all developmental genes, whose expression is altered by test compounds (Shinde et al., 2017[20]). Although large validation studies are still required, several developmental toxicants, e.g. thalidomide and valproic acid, have been successfully differentiated from negative control compounds (Meganathan et al., 2012[16]; Waldmann et al., 2014[27]). 

Much progress has been achieved in analyzing disturbed developmental processes. However, it still remains challenging to differentiate stem cells to adult cell types that closely resemble the corresponding mature cell type in vivo (Godoy et al., 2015[8]; Cameron et al., 2015[4]). The authors of the present review describe why this is so difficult, using stem cell derived hepatocyte-like cells as an example (Sachinidis et al., 2019[18]). Problems are due to incomplete endoderm patterning (Zorn, 2008[29]; Gordillo et al., 2015[9]) and to still suboptimal protocols to trigger the final differentiation of hepatoblasts to mature hepatocytes. 

Stem cell-based alternative test methods offer powerful tools to analyze developmental toxicity *in vitro*, but there is still a long way to go until the techniques are ready for routine use and to replace animal studies in pharmacology and toxicology. 

## Conflict of interest

The author declares no conflict of interest.
